# Proceedings of Medical Societies

**Published:** 1870-10

**Authors:** Z. C. McElroy

**Affiliations:** Zanesville, Ohio


BUFFALO

### Medical and Surgical Journal.

VOL. X. OCTOBER, 1870. No. 3.

### Original Communications.

ART. I.—*Proceedings of Medical Societies.—Report of Committee on New Remedies, to the Muskingum County, Ohio, Medical Society for October*, 1870. By Z. C. McElroy, M. D., *Zanesville, Ohio.*

Veratrum Viride as antidote for over-dose of Opium. Possibilities and impossibilities in t r peutics; flooding the system with tepid water as a therapeutic measure, with report of case; changes in the telluric forces and results in organic life, &c., &c.

One of the most noticeable announcements of a new use of an old remedy during the past month, is that of Veratrum Viride in large doses, for over doses or so-called poisoning from over doses of opium. This use of Veratrum is, however, only backed up by one clinical case. Yet it is an empirical fact that opium is the so-called antidote to the so-called poisoning of Veratrum Viride; and, *cæteris paribus*, they must be mutually antidotal. They both operate by diminishing the velocity of chemical changes in the tissues. Opium generally without elimination, and Veratrum Viride in large doses with very active elimination. The number of deaths, recorded and unrecorded, from opium are legion; while no solitary record of a case of death from Veratrum Viride has been found after very careful search of text-books and medical journals. And these facts cover explanations of the *modus operandi* of many so-called poisonous agents. Deaths are in several instances recorded from Aconite and Gelseminum, agents which retard motion, or chemical changes in the tissues, without elimination. Death from chloroform, and all the so-called anæsthetics, are far too numerous, and will most likely and unavoidably occur from chloral, for like reasons, that it is not followed by elimination.

The very frequency of sick stomach following the administration of opium, and more particularly in cases with so called idiosyncracies forbidding its use, is the exact reason why deaths from opium are not more frequent in general practice. Looked upon ordinarily as a calamity, it is rather the surety that fatal results shall not follow its use under these circumstances.

The recognition that there are in practical therapeutics, probabilities and improbabilities, possibilities and impossibilities, will do something towards evoking order out of existing confusion; for these have an undoubted existence in any scheme of scientific medication. No impossibilities have ever been recognized by the profession in any age, though impossibilities are numerous and prominent. The possibility that every form of so-called disease will ultimately be controlled and cured by therapeia is, perhaps, very nearly universally recognized by the profession at the present moment; though disappointment have closely followed practice and experiment for twenty-five centuries.

Thus, any claim set up that a particular medicine, or plan, or scheme of medication has ever cured a case of so-called fever, must be regarded as doubtful; even in the so-called periodical fevers, for relapse, after so-called cures is the rule, to which, however, there are occasional exceptions. The power to interrupt the phenomena of periodical fevers possessed by Quinia, and certain other agents, as arsenic, no one acquainted with the facts will question, or doubt; but the immediate return to so-called health is the exception and not the rule, circumstances being equal in all cases. The admission of improbabilities and impossibilities as factors in practical therapeutics will get rid of a vast amount of needless medication, some of which cannot be otherwise than damaging. Fixing some limitations to probabilities, and possibilities will go a long ways towards reducing practical therapeia to scientific order.

The law to which your attention was called in last month's report, that "function is form or structure speaking," is, so far as your Committee can see, as absolute as the law of gravity itself; and must be a prime factor in any scientific pathology. The number of so-called functional diseases or states, will ultimately be found very small, if indeed, as at present understood, they do not wholly disappear before scientific scrutiny of structure. And the non-recognition of impossibilities and improbabilities by the profession is the foundation of the gigantic traffic in patent medicines in our times.

One of the most remarkable summaries for our guidance in the remedial management of any pathological state is that of Dr. Thomas Inman, of Liverpool, in the *Medical Mirror*, published in that city. Referring to Phthisis and general debility, he says his favorite formula is, "Keep the stomach for food; the rectum for physic; and the skin for oil."

The system of extra stomachic medication inaugurated by the hypodermic syringe, and lung inhalation, is still in its infancy, and will doubtless be extended to the remedial management of more states than consumption and general debility. Were it not for the prejudice in our own country against the use of rectal syringes, a very large amount of discomfort and actual pain could be wholly avoided, reference being had to the so-called pathological states of the genetalia, of both male and female. A really good hypodermic syringe, skilfully used to relieve pain, is rarely objected to by any person, after the first time it is used. A flexible rubber syringe is kept in my own water-closet, with a jar of water, all the year round, save in freezing weather; and hardly a day passes during which it is not used;—result, no piles, constipation, or any of their sequelæ in my person.

Your attention is invited to the use of tepid water, which, for the want of a more suitable name, I designate "flooding the system with water." Its purpose is to fill the blood vessels, quicken the circulation, and all the molecular operations of the body, and as it were, washing out of it effete matter. Its use may be illustrated by the following case, treated since our September meeting:

September 7.—Mrs. Mosgrove, aged 28, mother of three children: A delicate woman in every respect; has been under treatment for a week or more for general debility, using agents increasing motion in the interest of repair; but is still almost without appetite, and has to force into her stomach most of what she does eat, against her taste in the matter; has had backache and headache a great deal; complains of great weakness, and is now sick at the stomach. Her color is not good; pulse small and theady; surface cool; pupils large, temperature barely natural; is pale and careworn; face wrinkled and dry; but she is up most of the time caring for her family.

My conclusion was that she had not fluids enough in her body to carry on the molecular changes necessary for her to feel well. The ideal may be differently expressed by saying that her blood was too thick to circulate. I told her to bring me a pitcher of warm water, holding two quarts, with a tumbler and slop-bucket. She promptly brought a two-quart tin pan as full as she could carry it, and a pint bowl, and then inquired what I was going to do with it. On being told that it was for her to drink, and that she could drink this pan, and another full, despite her bad feelings, she burst into a laugh, and declared she never could drink one bowl full. I remarked cheerily to her that she would be surprised at how much she could drink, if she tried, and would be more surprised and gratified at the relief she would get from it. The first bowlful was swallowed with much difficulty; the second more readily, and no further trouble was encountered in getting all of the first panful down, which was something more than three pints. Her pulse rose in volume considerably during this time, and she felt uncomfortably full. Noticing that her clothing fitted closely, she was requested to remove it, which she did, and felt more comfortable. The second panful, at her request, was made quite warm, and after some delay it was all swallowed, the total quantity something over three quarts. About two pints were returned by spitting out mouthfulls, and partial vomiting during the time. All the time she was taking it she was urged not to vomit it up. Her face had now become quite ruddy, and glowing with color, pulse full and bounding; sick stomach, as well as headache and backache, all gone; surface warm and moist. As it was near dinner time I left her, requesting her not to throw up any more if she could prevent it, but when the sense of fullness subsided, to eat her dinner, and to continue her medicine. She was not seen again till late in the afternoon of next day. On opening the door to admit me, she burst into a hearty laugh, and asked me if I had come to give her more water. In answer to my inquiries, she stated that a good while after I left her yesterday she threw up a great deal, felt better, eat a hearty dinner, and had not felt so well at any time during the last five years as she had done since, and did not care a fig for any doctor that day. And her improvement has been permanent, for when last seen, near the close of September, she was quite well for her. I should have mentioned that the great bulk of the water retained appeared at the bladder during the night.

This is by no means a solitary case. Your whole time at this meeting could be occupied in detailing cases more or less similar.

My mental conceptions leading to this use of water are, that the body is like the physical world in the midst of drougth, and flooding it with water is followed by the same delightful effects as rain in the physical world. And bearing this idea in the mind, but little difficulty can be encountered in determining when it is indicated as a therapeutic measure. And as a measure so potent for good, without any danger of doing mischief, it is the peer of Chloral, Chloroform and the hypodermic syringe.

Your Committee desire to call your attention to the remarkable disturbance of the telluric forces accompanying, and succeeding the storm of wind and rain on Friday afternoon, 9th Sept., ult. The effects of this disturbance began to be felt in organic life within three days, and by Thursday, 15th, a very large proportion of our population were more or less affected; some by what they called bad colds; sneezing, coughing, and large discharges from the mucous surfaces. In others, by sore throat, swelling of the glands about the neck, preceded by chilliness, and followed by mild febrile paroxysms, with headache, slight nausea, or positive sick stomach, capricious appetite, fetid breath, and in many instances bowel complaint with bloody discharges, particularly in children. In others, rheumatic pains about the back and loins, muddy complexions, and more or less fever. In others, again, erratic pains about the chest, asthmatic difficulties, with more or less difficulty in breathing. In other cases, again, erruptions on the cutaneous surfaces, or abcesses, more particularly in children, and in some children phenomena closely resembling cholera infantum. The disturbances of the reparative processes were very conspicuous, and the amount of discomfort in consequence very large.

More than half the people, of all ages, with whom I came in contact on the 19th, 20th and 21st days of the month were more or less unwell from one or more of these several results. Not many were confined to the house, or bed; and comparatively few of the total number disabled sought professional advice, though a good many sought relief from domestic remedies of various kinds. I suffered severely myself with the catarrhal phenomena, had frequent sneezing, some sore throat, cough and fever. I could neither read nor study, with any comfort, though my appetite scarcely failed me, except that I wanted dainties and knick-knacks, rather than good wholesome protoplasm. The only death occurring in my practice during the past season, took place on Friday evening, 16th ult., hastened as I believe by these changes in the physical forces, though probably not due to them wholly, for the patient was under treatment for the sequalæ of scarlet fever, and was in a very precarious state with unfavorable prognosis.

A very brilliant display of the aurora borealis occurred on Saturday evening, the 24th. The 22d and 23d days of the month were unusually warm, the themometer ranging between 80° and 90°. But after the aurora the temperature sunk down, ranging between 70° and 80° the remaining days of the month, with a noticeable increase in the disturbances of organic life, and particularly of periodic types. Some rain fell on the 26th, 28th, 29th and 30th. Fires in houses became very comfortable during many of these days.

These disturbances of the physical forces and organic life are facts, and facts having some relation to each other beyond all doubt; for they have been thus recognized and accepted as cause and effect from the remotest antiquity; but they have remained unexplained until our own times. More conspicuously now than ever before is man, or the physical body of man, studied through the things of earth which lead up to it; and a very recent writer* declares that whoever does not do so, "will enter in a labor which, if not a sorrow to himself, will assuredly be sorrow and vexation of spirit to others."

There were not wanting, however, in the not very remote past, far-seeing men, who obtained mental glimpses of what science now demonstrates in this connection. Thus, Mulder, a German physiologist, declared, half a century since, that material, form and

* Maudsley, Gulstonian Lectures, 1870.

function were the unities of organic life, and were always and inseparably associated. Accepting this as true, and science demonstrates it physically now, there was still a mystery in regard to function. But modern science clears this up by showing that force is stored up in the organs and tissues themselves; that function is performed at the expense of substance; that for each mechanical result, as the heaving of the chest, contraction of the heart, or other muscle; or for each act of vision, hearing, touch, taste or smell; or for each emotion, as loving, hating, etc.; or for the maintaining of the temperature at, above or below the normal standard; or for each chemical metamorphosis of food through any of its stages to solid tissue; or each act of intellection there is, for each, and all, changes of matter; that is, for each act of a living body, portions of compex matter have been reduced to simpler chemical states; and finally, that for the evolution of any life phenomena, physiological or pathological, matter must be worked up with a definite form; and chemical changes must take place in the molecular structure of the form, as an indispensable condition for the performance of a function; and as form is changed or lost, so is function, and in any given case, to the extent of the change or loss, forever suspended.

Function is, then, form or structure speaking, and is therefore the basis of all pathology. Is function annihilated, as in the case of vision; then the molecular form of the eye has been lost. Is it changed; then behind the change, alterations of molecular motion or form will be found. Studying the human body through the things of earth which lead up to it, the results of the changes in the physical forces, culminating in the storm of Friday, 9th Sept., become intelligible. Were organic forms or structures interfered with? Let the account of changed functions submitted to you a few moments since reply. Let the increased mortality in our midst since then, and it has been very large, have its due weight in forming conclusions. Stated differently, the earth sickened, and its organic life sickened with it. Nor were these influences confined to human life, nor animal life; they extended even to inanimate machinery. The foliage of the earlier deciduous trees prematurely fell, so that many of them only presented to vision naked branches, long before the close of the month. And the premature appearance of the magnificent autumn tints peculiar to our forest trees are evidence that some unusual influences have been at work. And one of the superintendents in the large railway shops located here tells me that the press on them for repairs is greater now than at any time during the past year. Watches and clocks of the finest construction, which ran evenly with each other during the summer, present much variation in their seconds now.

But for the disturbances in the physical forces during the greater part of last month these things would not have occurred as they did and when they did. It would only be dodging the question to say that these changes were in consequence of the season. It is these disturbances in the physical forces which make seasons. Such a disturbance occurring in the middle of June, extending through a similar length of time, would occasion, in addition to these wide-spread and immediate results, a partial famine by arresting, or blighting vegetable growth, upon which all animal life is ultimately dependent for the material with which to construct their tissues.

